# The Crystallization of Disordered Materials under Shock Is Governed by Their Network Topology

**DOI:** 10.1002/advs.202300131

**Published:** 2023-04-28

**Authors:** Longwen Tang, Pratyush Srivastava, Vijay Gupta, Mathieu Bauchy

**Affiliations:** ^1^ Physics of AmoRphous and Inorganic Solids Laboratory (PARISlab) Department of Civil and Environmental Engineering University of California Los Angeles CA 90095 USA; ^2^ Department of Mechanical and Aerospace Engineering University of California Los Angeles CA 90095 USA

**Keywords:** crystallization, molecular dynamics simulation, shock, topological constraint theory

## Abstract

When the shock load is applied, materials experience incredibly high temperature and pressure conditions on picosecond timescales, usually accompanied by remarkable physical or chemical phenomena. Understanding the underlying physics that governs the kinetics of shocked materials is of great importance for both physics and materials science. Here, combining experiment and large‐scale molecular dynamics simulation, the ultrafast nanoscale crystal nucleation process in shocked soda‐lime silicate glass is investigated. By adopting topological constraints theory, this study finds that the propensity of nucleation is governed by the connectivity of the atomic network. The densification of local networks, which appears once the crystal starts to grow, results in the underconstrained shell around the crystal and prevents further crystallization. These results shed light on the nanoscale crystallization mechanism of shocked materials from the viewpoint of topological constraint theory.

## Graphical abstract

Formation of nanoscale crystal in shocked soda‐lime glass is found in both experiments and million‐atoms molecular dynamics simulation. Topological analysis reveals that the connectivity of the atomic network governs the propensity of nucleation in the shocked oxide glass. The results shed light on the nanoscale crystallization mechanism in an off‐stoichiometric system.

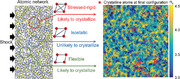

## 1 Introduction

Under the shock load, the material region near the shock wave experiences extreme stress and temperature conditions. For example, the shocked material can experience pressure in tens of GPa, with an attendant temperature of several thousand degrees.^[^ ^1^, ^2^ ^]^ During this process, the material can undergo physical and chemical changes via plastic deformation,^[^ ^3^ ^]^ solid–solid phase transition,^[^ ^4^, ^5^ ^]^ and chemical reaction.^[^ ^6^, ^7^ ^]^ The physical and chemical behavior under shock usually shows an anomaly compared to the one under normal conditions.^[^ ^7^ ^]^ A typical example is the crystallization of silica under shock, which forms a dense stishovite phase that is rare in nature since it is in a metastable state under a normal environment. As a result, this dense phase usually exists in meteor craters on planets.^[^ ^8^, ^9^ ^]^ Understanding the crystallization mechanism under shock is of critical importance in materials science and geophysics (e.g., developing impact‐resistant materials and understanding meteorite impact).

When a liquid relaxes below its melting temperature, crystallization can occur according to thermodynamics. Before forming a macroscopic crystal, the clusters of crystalline atoms nucleate and grow to reach a sufficiently large size, which overcomes the free energy cost of creating a new surface by the free energy gain of forming the stable crystal.^[^ ^10^ ^]^ Many studies have been conducted to understand the mechanism that controls the nucleation process of various materials.^[^ ^11^, ^12^, ^13^, ^14^, ^15^ ^]^ Most of the crystallization research focuses on stoichiometric composition, where the composition of the crystal phase is the same as the parent glass. When the compositions of participated crystal and parent glass are significantly different, the crystallization process can be much more complicated due to the evolution of the residual glass composition.^[^ ^16^, ^17^, ^18^ ^]^ This type of crystallization is also called off‐stoichiometric (or nonstoichiometric) crystallization. In addition, well‐controlled off‐stoichiometric crystallization is the key to making the glass‐ceramics with the desired physical properties.^[^ ^19^, ^20^ ^]^

In recent decades, various advanced experimental approaches have been employed to study the kinetics of crystal nucleation in disordered materials.^[^ ^21^, ^22^, ^23^, ^24^ ^]^ Due to the limitation of the spatial and temporal resolution of most experimental methods, they can hardly provide true microscopic insight into the nucleation process of glasses,^[^ ^15^ ^]^ which usually occurs within a few nanoseconds at a small length scale (few nanometers). As a promising alternative approach, molecular dynamics (MD) simulations have dramatically improved the fundamental understanding of the nucleation mechanism (e.g., evidence of the two‐step nucleation mechanism,^[^ ^25^ ^]^ verification of classical nucleation theory,^[^ ^26^, ^27^ ^]^ formation of metastable phase before the nucleation,^[^ ^28^, ^29^ ^]^ see ref. [15] for more examples). Note that because of the existence of thermal fluctuation, the local composition and structure around the nucleus are varied during the nucleation process. Recent simulations show that the local compositional and structural fluctuation can play an important role in the nucleation process,^[^ ^30^, ^31^ ^]^ even for the stoichiometric composition. As the composition of residual liquid keeps changing during the off‐stoichiometric nucleation process, its compositional fluctuation can be much more dramatic compared with the stoichiometric one. Nevertheless, the evolution of the local composition and structure, as well as its impact on the off‐stoichiometric nucleation process, remain largely unknown.

Here, by employing a laser‐generated flyer plate impact test and large‐scale MD simulation, we investigate the off‐stoichiometric nucleation process in soda‐lime silicate glass (SLG) under shock. The formation of the nanoscale crystal phase in the soda‐lime silicate glass is observed by both experiments and MD simulations. Interestingly, we find that the crystal growth arrests when its size reaches around 3–4 nm, resulting in the breakdown of power‐law size distribution. From the viewpoint of topological constraint theory, we show that the propensity of crystallization in soda‐lime silicate glass is governed by the local connectivity of the atomic network. Upon nucleation, the local atomic network becomes denser in the central part of the nucleus, while the outer layer of the nucleus forms the underconstrained shell, which prevents the further growth of the crystal.

## 2 Results and Discussion

### 2.1 Experimental and Numerical Evidence of Nanoscale Crystallization

**Figure 1:**
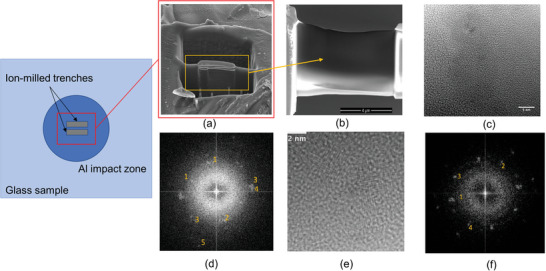
SEM images showing a) TEM sample preparation using FIB from the impact site of the recovered sample. The two depressions are the FIB milled trenches in the Al impacted zone on the glass sample. The shock‐loaded glass material “bridge” between the trenches is then lifted off from the bulk using TEM mount and then thinned to <100 nm thickness, shown in (b). Stishovite formation in soda‐lime glass sample, subjected to 22 GPa shock pressure for 8 ns: c,e) high‐resolution TEM images from two different regions of the sample. f),d) Their corresponding FFT analysis with diffraction spots marked (Table S1, Supporting Information).

To investigate the crystallization mechanism of SLG under shock, we use a table‐top laser‐generated flyer plate impact setup to impose the shock load on the SLG (71.2%SiO_2_•13.9%Na_2_O•8.2%CaO•4.4%MgO•1.9%Al_2_O_3_•0.4%K_2_O) plate. SLG plate is impacted by an Al flyer plate, which in turn is generated and propelled by a spatially top‐hat Nd:YAG laser pulse. The peak stress is measured during the impact process using a state‐of‐the‐art photonic Doppler velocimeter (PDV). As shown in **Figure** **1**a, after the impact test, TEM samples are prepared from the center of the impacted region of the recovered sample by FIB micromachining. Then, TEM samples shown in Figure 1b are examined using an FEI‐Titan scanning/transmission electron microscope (S/TEM) at 300 kV (see the Experimental Section for more details of experimental setup). Interestingly, we find the existence of nanocrystallization through atomic‐scale imaging and fast Fourier transform (FFT) analyses. As shown in Figure 1c–f, the typical size of nanocrystals in the selected locations is around 3–4 nm. Then we compare the interplanar spacing of the marked diffraction spots from the FFT analysis with that of the stishovite, SiO_2_, and Pt. As illustrated in Table S1 (Supporting Information), we find there is only a negligible difference between the interplanar spacing of stishovite and the crystal observed in this study, indicating stishovite formation during the shock process.

**Figure 2:**
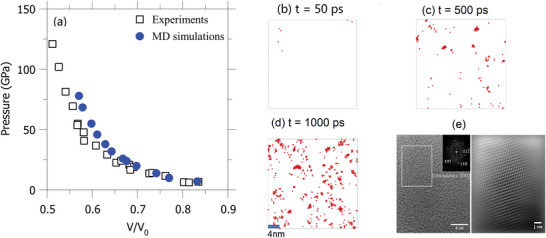
a) Comparison between MD and experimental results of shock Hugoniot for SLG. MD results are obtained at *t* = 100 ps. b–d) Snapshots of crystal Si atoms at selected time *t*. e) HRTEM analysis of a single Stishovite crystal. The left shows the FFT of the region enclosed by the white rectangle, which shows the Stishovite phase that is oriented to the [−111] zone axis. The right shows Fourier filtered image of the region enclosed by the white rectangle in left.

We then investigate the nanoscale crystallization of shocked SLG through MD simulations. To overcome the expensive computational cost of traditional nonequilibrium molecular dynamics simulation,^[^ ^32^ ^]^ we employ the multiscale shock technique (MSST) to mimic the shock on the SLG that consists of ≈1 million atoms.^[^ ^33^ ^]^ The initial SLG sample is obtained from the conventional melt‐quenching procedure (see the Experimental Section for more details). We first validate the MD simulations by comparing them with experimental data. To this end, a series of shock velocities are selected to achieve different pressures and temperatures. As shown in **Figure** **2**a, we observe a good agreement between numerical (obtained at 100 ps) and experimental Hugoniots (i.e., compression–pressure relationship),^[^ ^34^, ^35^, ^36^ ^]^ which suggests the numerical method used in this study is able to offer a realistic description of the thermodynamics of SLG under shock conditions.

To visualize the crystallization process, we employ the shape matching analysis(see method section for more details) to MD simulation results to distinguish the atom that belongs to the crystal (i.e., crystalline atoms).^[^ ^37^ ^]^ Here, we focus on the MD simulation results corresponding to relatively high shock velocity (i.e., 7.8 km s^−1^), where the crystallization occurs within 1 ns under high temperature (i.e., ≈3300 K) and pressure (i.e., ≈60 GPa). Note that the relatively high pressure and temperature are selected to expedite the crystallization process (see the Supporting Information for more discussion), which helps to reproduce the crystallization without exceeding current computational capability. Figure 2b–d shows crystalline atoms (marked as red) at different times after the shock (noted that we only show the Si atom in the snapshots). Once the shock load is imposed, the SLG is suddenly compressed under elevated temperature within 10 ps. Then, some isolated crystal‐like local structures randomly form and disappear, as illustrated by the isolated single crystalline atom in Figure 2b at 50 ps. After several hundreds of ps, some nuclei survive and gradually increase (see the small clusters in Figure 2c). Finally, we observe several large crystals with a typical size of 2–4 nm at *t* = 1 ns, which agree well with experimental observation as shown in Figure 2e (also see Figure 1c–e).

### 2.2 Kinetics of SLG Crystallization

**Figure 3:**
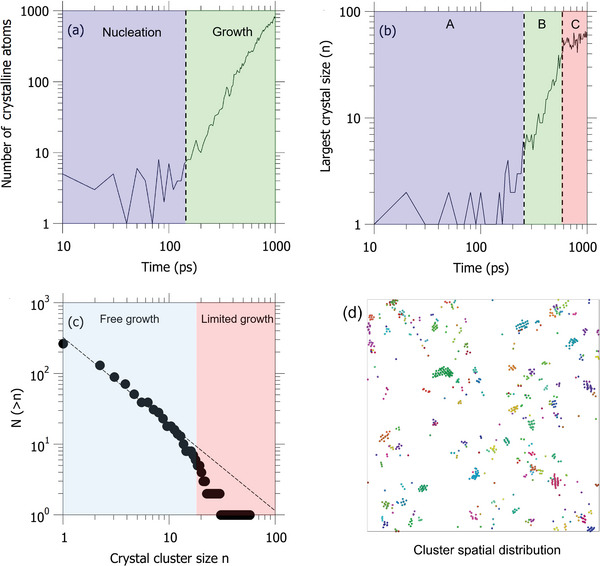
Top figures show the evolution of a) the total crystal‐like Si atom and b) the largest crystal size (i.e., the largest number of crystal‐like Si atoms in the crystal), where A, B, and C represent different stages for largest crystal formation. c) The cumulative distribution of crystal size at *t* = 1 ns. d) Snapshot of crystal cluster distribution at *t* = 1 ns, where clusters are colored to distinguish each other.

We now investigate the crystallization dynamics of the shocked SLG. **Figure** **3**a shows the evolution of the total number of the crystal Si atoms. We observe that the total number of crystalline atoms vibrates around 4 at the initial stage, which indicates the nucleation stage, where nuclei form occasionally and quickly dissolve. However, after 150 ps, some nuclei successfully survive and reach the critical size (i.e., the size that is thermodynamically stable), which results in a power‐law increase of crystalline atoms (see the green in Figure 3a). To further illustrate this process, we plot the largest size of the crystal (i.e., the number of Si atoms in a single crystal) as the function of time (Figure 3b). At the nucleation stage (cyan region), the largest crystal can only contain two Si atoms until a nucleus successfully grows up to reach the critical size (i.e., containing five Si atoms and equivalent to ≈10 Å), which is comparable with the critical size for pure silica liquid under high temperature and pressure.^[^ ^4^, ^27^ ^]^ Then, the size of the largest crystal increases explosively in a similar power‐law fashion shown in Figure 3a (green region). Interestingly, we find that the growth of the largest crystal stops after around 600 ps, as evidenced by the fluctuation of the largest size around 65 Si atoms in the red region.

Figure 3c shows the cumulative distribution of crystal size. For the crystal size range from 1 to 12, the crystal size follows a clear power‐law distribution, which has been widely reported in nature complex systems.^[^ ^38^ ^]^ However, the crystal size distribution gradually departs from the main curve (the dashed line) for large crystal size (red region). The anomaly of crystal size distribution comes from the unexpectedly small number of large crystals. This result suggests that, in contrast to the free growth of crystals in stoichiometric liquids (e.g., silica crystallization),^[^ ^4^ ^]^ further crystallization of large nuclei is limited. Consequently, we observe the spatially distributed isolated nanocrystals with a typical size of 2–4 nm (see Figure 3d).

### 2.3 Propensity of Crystallization

**Figure 4:**
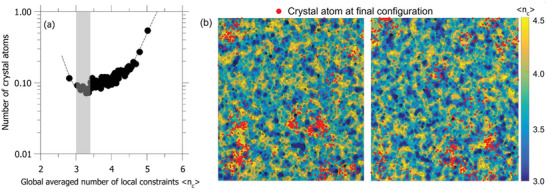
a) The number of crystalline atoms at *t* = 1 ns as a function of the global averaged number of local chemical constraints 〈*n*_c_〉 at *t* = 150 ps. Each of the data points is averaged over 2000 Si atoms (as sorted in terms of increasing values of $\left\langle n_{c}^{i}\right\rangle$). b) Contour maps show the spatial distribution of 〈*n*_c_〉 at *t* = 150 ps. Red circles mark the positions of atoms that will crystallize before *t* = 1 ns.

We then investigate the underlying physics governing crystallization propensity in shocked SLG. In this study, we extend the classical topological constraint theory to the local situation.^[^ ^39^ ^]^ To this end, we calculate the global averaged number of local chemical constraints $\left\langle n_{c}^{i}\right\rangle$, which represents the connectivity of the atomic network within the local region of atom *i* at *t* = 150 ps (see Experimental Section for more details). Note that the local region is defined as a sphere of radius *r* = 5 Å centered on atom *i*. Therefore, we can evaluate the local connectivity of the atomic network by comparing the global averaged number of local chemical constraints $\left\langle n_{c}^{i}\right\rangle$ with the atomic degrees of freedom (i.e., 3). When $\langle n_{c}^{i}\rangle >3$, redundant constraints emerge in the network and result in some internal stress. When $\langle n_{c}^{i}\rangle <3$, some floppy modes occur in the network due to the lack of constraints. The optimal condition is achieved when $\langle n_{c}^{i}\rangle =\;3$. Based on the calculated $\left\langle n_{c}^{i}\right\rangle$, the local atomic networks can be classified as (i) flexible if $\langle n_{c}^{i}\rangle <3$, (ii) stressed‐rigid if $\langle n_{c}^{i}\rangle >3$, and (iii) isostatic if $\langle n_{c}^{i}\rangle =\;3$. Then we calculated the number of crystalline atoms (only Si atom) *N^i^* within the local region of atom *i* at *t* = 1 ns. **Figure** **4**a shows the number of crystalline atoms at the final stage (*t* = 1 ns) as a function of the local average number of chemical constraints at the nucleation stage (*t* = 150 ps). For statistical averaging purposes, each of the data points shown in Figure 4a represents the averaging of *N^i^* and $\left\langle n_{c}^{i}\right\rangle$ over 2000 Si atoms (as sorted in terms of increasing values of $\left\langle n_{c}^{i}\right\rangle$). Interestingly, we find the atoms within the isostatic network exhibit the lowest propensity of crystallization (see the grey region in Figure 4a). Meanwhile, we observe a higher probability of forming crystals in both flexible and stressed‐rigid networks.

We now explain this propensity from the viewpoint of topological constraint theory. When the local network is flexible (i.e., $\langle n_{c}^{i}\rangle <3$), the local network exhibits more flexibility since there are more atomic degrees of freedom than constraints.^[^ ^40^ ^]^ Thus, atoms can easily reorganize themselves to form the minimum energy structure, which promotes crystallization. In contrast, for the local stressed‐rigid network (i.e., $\langle n_{c}^{i}\rangle >3$), residual constraints can hardly release the stress by adjusting themselves since there are more constraints than atomic degrees of freedom. As a result, overconstrained network features non‐ignorable internal stress,^[^ ^41^ ^]^ which acts as a driving force that facilitates the system relaxation towards lower energy states and enhances the thermodynamic propensity for crystallization. Therefore, the local isostatic network exhibits the highest resistance to crystallization.

To further illustrate this, we draw the contour plots of $\left\langle n_{c}^{i}\right\rangle$ of selected slices at the final configuration (see Figure 4b). The red point on the contour plots corresponds to the atom that will crystallize by the end of the simulation. The average $\left\langle n_{c}^{i}\right\rangle$ for crystal atoms is 4.23 at 150 ps. These atoms are more likely to overlap with the highly stressed‐rigid region ($\langle n_{c}^{i}\rangle >4$) and bypass the isostatic region, which is consistent with the trend from Figure 4a. However, we only observe a few red points on the flexible region due to its low proportion in the contour map. This result suggests that although the flexible region is more likely to form nuclei than the isostatic one, a large crystal is unlikely to appear in this region. Moreover, we observe that the distributions of local chemical constraints and atoms that will crystallize are strongly spatial heterogeneous, contrasting to the homogeneous hypotheses in the mean‐field model at the nanoscale.

### 2.4 Arrest Mechanism of Crystallization

**Figure 5:**
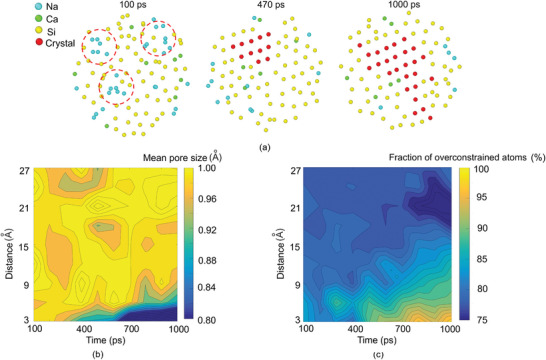
a) Snapshots of a large crystal formation process at select time *t*. b,c) Plot of the mean pore size and the fraction of overconstrained atoms as the function of time and distance from the center of a large crystal. The red dashed circles represent the glass modifiers concentration region before the crystallization.

To understand the microscopic mechanism of the arrest of crystallization at a late stage, we now focus on the crystallization process of a large crystal. **Figure** **5**a shows snapshots of atomic structure within the selected region where the crystal eventually forms. For better visualization, O atoms are not displayed in snapshots. At the nucleation stage (100 ps), we observe that a Si‐rich region is surrounded by some Na and Ca atoms. It indicates that the spatial heterogeneity of network connectivity (illustrated in Figure 4b,c) may stem from the chemical heterogeneity. After a few hundred picoseconds, a small crystal nucleates in the central Si‐rich region and reaches the critical size. For stoichiometric liquid, the chemical composition remains unchanged during the crystallization process. However, since the SLG crystallization is off‐stoichiometric, we find that most Na atoms gradually diffuse into the ambient uncrystallized liquid, as evidenced by the disappearance of Na‐rich regions circled in the left part of the plot. Then, the crystal continues to grow along the nearest Si‐rich region and finally forms the crystal shown in the right part of the plot.

To explain this behavior, we calculate the mean pore size in the spherical shell centered around the centroid of the selected crystal (see the Experimental Section for more details). Figure 5b plots the mean pore size as the function of the distance (i.e., the distance between the point on the shell and the centroid of the selected crystal) and time. Each point on the contour plot is averaged over the six largest crystals to obtain statistically meaningful results. We observe no clear trend of mean pore size distribution at the nucleation stage (i.e., around 100 ps). However, the dense phase closest to the initial nuclei gradually forms at 400 ps, accompanied by an incompact region around it. The existence of a dense atomic network prevents the permeation of other atoms. In other words, more driven force is needed for Na and Ca atoms to diffuse into this dense network. As a result, the increase of energy barrier for Na to diffuse into the Si‐rich region explains the unusual directional diffusion behavior of Na (i.e., Na atoms diffuse into a high concentration region).

The direct effect is the formation of Na‐ or Ca‐rich regions around the crystal. Since Na and Ca atoms act as network modifiers and depolymerize the atomic network,^[^ ^42^, ^43^ ^]^ the number of topological constraints in this region is expected to decrease. Figure 5c plots the fraction of overconstrained atoms (i.e., number of constraints > 3) as the function of the distance and time. Indeed, we observe a clear underconstrained region located at a distance of around 23 Å from 800 ps to 1 ns. Since atoms close to the isostatic state are less likely to form the crystal, this region act as the covering layer of the crystal and prevents its further growth. As a result, we observe isolated nanocrystals with a typical size of 2–4 nm (see Figure 3d). Overall, these results reveal two competing mechanisms: the network's densification in the core of nucleation and the decompaction in the outlier of nucleation. The former mechanism creates the overconstrained region where the crystal can grow, while the latter mechanism forms the underconstrained shell that prevents further crystallization. Once the driving forces of these two mechanisms reach equilibrium, the crystallization process stops and shows a constant in crystal size (see the red region in Figure 3b). Apart from soda‐lime silicate glass, the mechanism discovered in this study may also be applicable to other silica‐rich glasses that contain minor glass modifiers such as K_2_O, Al_2_O_3_, and Li_2_O. Additional investigations are necessary to validate the universality of the observed mechanism. As shown in Figure 5, the final crystal size in soda‐lime glass is primarily determined by the formation of an underconstrained shell around the crystal, rather than by the nucleation rate or crystal growth rate. As a result, the impact of pressure and temperature on the final crystal size is expected to be limited.

### 2.5 Effect of Stoichiometry, Pressure, and Temperature

**Figure 6:**
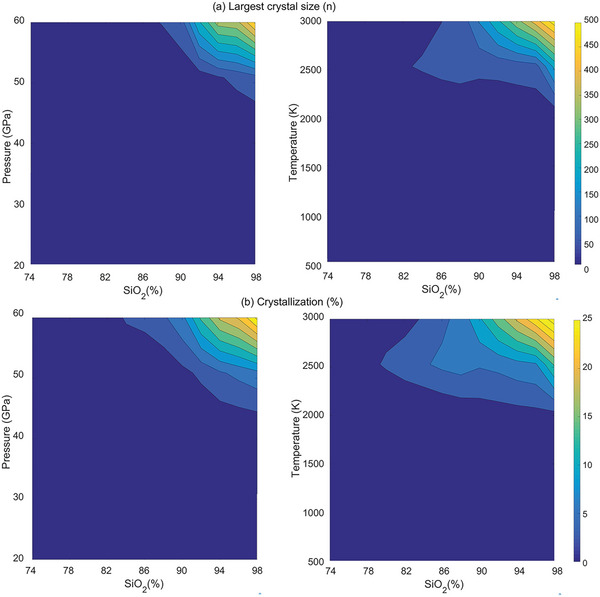
Counter plots of a) the largest crystal size and b) the percentage of crystalline in different compositions as the function of temperature (right) and pressure (left).

We now discuss the effect of stoichiometry, pressure, and temperature on the crystallization. To this end, we perform a series of shock simulations on the glasses with various compositions under different shock velocities. SiO_2(1−5_ *_x_*_)_•Na_2_O_(3_ *_x_*_)_•CaO_(2_ *_x_*_)_ glasses are generated by following the same melting‐quenching procedure. Note that we now focus on the crystallization in the small system (around 8010 atoms) within 1 ns. As shown in **Figure** **6**, it can be observed that, under identical temperature and pressure conditions, the crystal size increases with an increase in SiO_2_ content, while the opposite trend is observed when the SiO_2_ content is reduced. This phenomenon can be explained by the fact that low SiO_2_ content glasses have highly concentrated glass modifiers (i.e., Na and Ca), which can quickly form an underconstrained shell, preventing further crystallization. Conversely, high SiO_2_ content glass promotes larger crystal growth due to the reduced concentration of glass modifiers. In addition, previous experimental studies have shown that an increase in SiO_2_ content leads to an increase in the nucleus/liquid interfacial energy (also see refs. [16, 44] for lithium metasilicate),^[^ ^17^ ^]^ which, in turn, enhances the nucleation and crystal growth rates, resulting in the observed larger crystal size.

**Figure 7:**
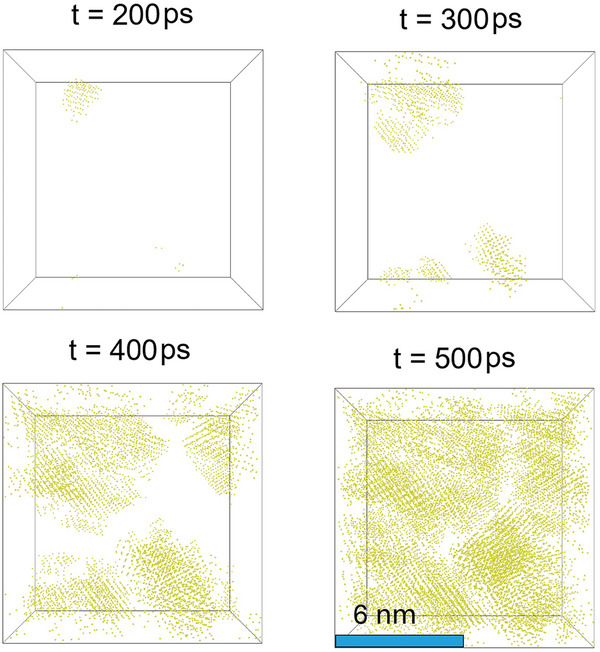
Snapshots of the spatial distribution of crystal Si atoms at selected times.

To investigate how stoichiometry influences the crystallization behavior of SLG, we conducted a large‐scale shock simulation on pure silica following the same procedure as in this study. The silica glass model we used consisted of 140 976 atoms, which is large enough to avoid finite size effects.^[^ ^4^ ^]^ A similar pressure (57 GPa) and temperature (3300 K) are achieved compared with the one obtained for SLG. **Figure** **7** shows that nucleation occurs before 200 ps, and large crystal grains (around 6–8 nm) form around 500 ps. This finding suggests that the nucleation and crystal growth rates in pure silica are significantly higher than those in SLG, which supports our previous analysis. In addition, we observe the typical crystallization behavior of polycrystals, where nuclei form randomly in space and rapidly grow until they reach their neighbors and form grain boundaries. Thus, the grain size in shocked silica is controlled by typical nucleation and crystal growth rates.^[^ ^45^, ^46^ ^]^ In contrast, the crystallization behavior in SLG is different. As shown in Figure 5, the growth of the crystal is prevented by an underconstrained shell made up of highly concentrated glass modifiers, which prevents further growth of crystal and leads to the arrest of crystallization before the crystal reaches its neighboring crystals. As a result, the crystal size of Stishovite in SLG is primarily determined by the chemical composition of the uncrystallized region.

## 3 Conclusion

In conclusion, we investigate the microscopic mechanism of the ultrafast crystallization process of shocked SLG. Through a laser‐generated flyer plate impact test and MD simulations, we observe the formation of nanoscale stishovite crystal after shock load. More importantly, our simulation results yield the typical crystal size that agrees well with that observed experimentally. Based on the topological constraint theory, we reveal that the fluctuations of local topological constraints govern the crystallization process. The flexible and stressed‐rigid local atomic network facilitates the reorganization of atoms and hence, exhibits a higher propensity to crystallization than the isostatic one. Moreover, we find that the arrest of crystallization is controlled by the competition between the network's densification in the core of nucleation and the decompaction in the outlier of nucleation. These results suggest that, unlike the widely investigated stoichiometric system, the off‐stoichiometric system exhibits a unique crystallization mechanism under shock. Our results shed light on the solid‐solid transition in an off‐stoichiometric system, which could have vast applications, e.g., understanding meteorite impact, designing ballistic resistant materials, and glass‐ceramics.

## 4 Experimental Section

### Plate Impact Experiment

**Figure 8:**
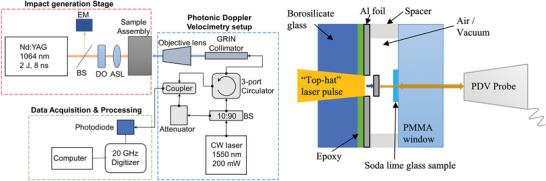
Schematic plots of punching of the flyer disc by the top‐hat laser pulse and measuring the flyer velocity and shock arrival at the sample's back surface from PDV.

**Figure** **8** shows the cross‐sectional view of the laser‐generated flyer plate impact setup.^[^ ^47^ ^]^ An Nd:YAG pulse laser with 2 J max pulse energy was used as the launch laser. The single‐shot output beam is 12 mm in diameter and 8 ns long. It is first converted to a “top‐hat” spatial profile and then focused onto a 1 mm diameter spot on the back surface of a 25 µm thick Al foil bonded to a transparent borosilicate glass window. As a result, a 1 mm diameter flyer disc was punched out and accelerated due to the absorbed optical radiation.^[^ ^48^ ^]^ The flyer reaches a maximum velocity of a few kilometers per second within 100 ns.

PDV was used to measure the particle velocity (see the Supporting Information for more details), *u*_p_ = 1.69 km s^−1^ at the sample's back surface, and the shock velocity, *u*_s_ = 5.19 km s^−1^ through the sample. Then, the compressive stress can be obtained as:*σ* = *ρ*_0_ *u*_s_ *u*_p_, where *ρ*_0_ is the initial density of SLG. Detailed setup and parameters of PDV can be found in the previous publication.^[^ ^47^ ^]^

### MD Sample Preparation

The SLG model (75%SiO_2_•15%Na_2_O•10%CaO) consists of 1 027 628 atoms and is randomly generated in a box at the beginning. Other minor components (e.g., MgO, Al_2_O_3_, and K_2_O) were not considered because of their relatively low content and similar role as glass modifiers. Note that boundaries in all directions are periodic. Then, the system was melted at 3000 K and zero pressure for 200 ps in the isothermal–isobaric (*NPT*) ensemble with a Nosé–Hoover thermostat to lose its memory for the initial position.^[^ ^49^, ^50^ ^]^ Next, the obtained liquid was cooled to 300 K with a cooling rate of 10^12^ K s^−1^, followed by additional relaxation at 300 K for 100 ps. The above melt‐quenching process was performed under zero pressure in the *NPT* ensemble. The timestep is 1 fs for all MD simulations. The interatomic interaction energy is described by Buckingham potential parameterized by Guillot and Sator,^[^ ^51^ ^]^ which yields a realistic atomic structure and bulk modulus of silicate melts under high pressure.^[^ ^51^, ^52^, ^53^ ^]^ Coulombic interactions were resolved using the particle–particle particle‐mesh (PPPM) method with an accuracy of 10^−4^.^[^ ^54^ ^]^ To avoid unrealistic high energy collision, the potential was splined with a Ziegler–Biersack–Littmark (ZBL) screened nuclear repulsion potential.^[^ ^55^ ^]^ All the simulations were conducted using the LAMMPS package.^[^ ^56^ ^]^

### Shock Simulation

To mimic the shock load, MSST was adopted to simulate the dynamics of a group of atoms embedded in the material that the shock wave travels through.^[^ ^33^ ^]^ In addition to the relatively low computational cost compared with nonequilibrium MD, this approach was found to reasonably describe the kinetics and thermodynamics of disordered systems under shock.^[^ ^4^, ^57^ ^]^ It should be noted that the shock wave instability occurs when the pressure is in the unstable region of hypothetical Hugoniot.^[^ ^33^ ^]^ Here, the procedure proposed by Evan J. Reed (i.e., simulating sufficient trial values of shock wave velocity) to handle the instability in shock simulation was followed (more details of MSST simulation are given in the Supporting Information).^[^ ^33^ ^]^ The computational cell mass *q* was selected as 40 amu^2^ Å^2^, which results in compression within 10 ps. The initial temperature reduction scale = 0.01 without artificial viscosity. The scale factor *β* was selected as 0.5 for improved energy conservation.

### Bond Order Parameter

To visualize the crystallization process, the Steinhardt parameter $q_{lm}^{s_{1}s_{2}}$ was first calculated,^[^ ^58^ ^]^ where *s*_1_ and *s*_2_ are type of central atom and neighbor atoms, respectively. Similar to previous work,^[^ ^4^ ^]^ this study only considered Si atom. Thus, the bond order parameter *q*_6_ for atom *i* at *l* = 6 can be written as

(1) $$q_{6}\left(i\right)\;=\frac{1}{N}\;\sum_{j}\frac{\sum_{m\;=\;-6}^{6}q_{6m}\left(i\right)\cdot q_{6m}\left(j\right)}{∥q_{6m}\left(i\right)∥\cdot ∥q_{6m}\left(j\right)∥}$$

where *N* is the number of atoms within 3.7 Å. Here, atom *i* was regarded as the crystalline atom if *q*_6_(*i*) > 0.75, which can precisely distinguish the stishovite from SLG liquid (see Figure S1, Supporting Information).

### Local Topological Constraint

In this study, the local average number of constraints was calculated rather than calculating the macroscopic average constraint number. In detail, the bond radial bond‐stretching and angular bond‐bending created by Si—O network were considered. The total number of Si—O bond *n*_s_, O—Si—O bond angle *n*_as_, and Si—O—Si bond angle *n*_ao_ in the selected region for atom *i* were first counted. Then, the $n_{c}^{i}$ was calculated as: $n_{c}^{i}=\frac{n_{s}+2n_{\mathrm{as}}+2n_{\mathrm{ao}}-2}{N}\;$, where *N* is the total number of Si and O atoms.

### Mean Pore Size

Mean pore size was used to evaluate the compaction degree of the atomic network. Atoms are regarded as spheres with corresponding ionic radius.^[^ ^59^ ^]^ For a given distance, the mean pore size was calculated within the spherical shell with a thickness of 3 Å by using the algorithm.^[^ ^60^ ^]^ Here, this study focused on the compaction degree of the Si—O network and glass modifiers (i.e., Na and Ca) were ignored. Hence, a pore can be defined as a region that lacks Si and O atoms.

## Conflict of Interest

The authors declare no conflict of interest.

## Supporting information

Supporting Information

## Data Availability

The data that support the findings of this study are available in the supplementary material of this article.
