# Supplementary material for: The Crystallization of Disordered Materials under Shock Is Governed by Their Network Topology
Source: Adv Sci (Weinh). 2023 Apr 28;10(20):2300131. doi: 10.1002/advs.202300131 (PMC10369245; doi:10.1002/advs.202300131)
Supplement: Supplementary file 1 — Supporting Information [file ADVS-10-2300131-s001.pdf]

ADVANCED SCIENCE
Open Access

Supporting Information

for *Adv. Sci.*, DOI 10.1002/advs.202300131

The Crystallization of Disordered Materials under Shock Is Governed by Their Network Topology

Longwen Tang, Pratyush Srivastava, Vijay Gupta and Mathieu Bauchy**

Supporting Information

The Crystallization of Disordered Materials Under Shock is Governed by their Network Topology

*Longwen Tang, Pratyush Srivastava, Vijay Gupta, * Mathieu Bauchy**

We perform additional MD simulations to determine the threshold to distinguish stishovite from soda lime glass. First, we generate a small soda lime glass sample (including 2996 atoms) following the same melt-quenching procedure. Then, we create a stishovite structure (including 1944 atoms) from the experimental data[1] and equilibrium it under 300 K for 500 ps. To mimic the realistic thermodynamical environment, the soda lime glass and stishovite are subjected to 3300K and 60 GPa. Figure. S1 shows distributions of order parameters of soda lime glass and stishovite. We find that $q_6 = 0.75$ can clearly separate two phases.

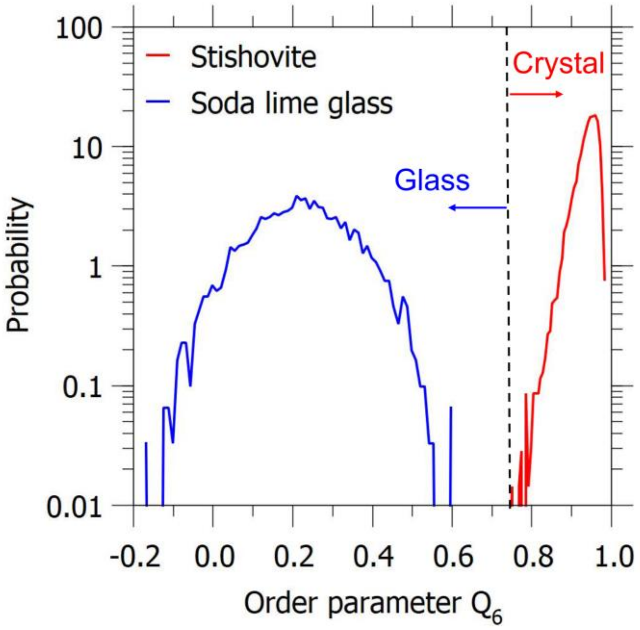

Figure S1. Distributions of order parameters of soda lime glass and stishovite.

Table S1. Interplanar (d) spacings measured from FFT analysis and its comparison with the known values for Stishovite, SiO_2, and Pt. from the literature. The index numbers refer to the ones shown in Fig 1.

#	d-measured (nm)	d-Stishovite (nm)	Int. (%)	Error (%)	d-SiO_2 (nm)	Int. (%)	Error (%)	d-Pt. (nm)	Int. (%)	Error (%)
1	0.29	0.2958	100	2.0						
2	0.25				0.2493	20	0.3			
3	0.24				0.2493	20	3.7			
4	0.23							0.2265	100	1.5
5	0.22	0.2248	22	2.2				0.2265	100	2.9
6	0.20				0.1917	100	4.3	0.1961	53	2.0
7	0.14							0.1387	31	0.9

In this study, the stress state (σ) of the sample was determined through the use of a Photonic Doppler Velocimeter (PDV), which measured the flyer velocity, particle velocity up at the sample's back surface, and the shock velocity (us) through the sample. The PDV was equipped with interferometric and launch laser beams, as well as other optical components, which are depicted in Figure 8. A probe consisting of a GRIN collimator and microscope objective was employed. The PDV beam, generated from a 1550 nm CW laser source (shown in Figure 8), was collimated to a diameter of 500 µm and then focused onto the front surface of the Al foil using an objective lens. The focused spot had a diameter of approximately 78 µm and a long Rayleigh length of 12 mm, which ensured that the flyer disc remained in focus throughout its flight distance of 500 µm before colliding with the SLG sample. The pulse and probe beams were accurately aligned using lasers to ensure that the probe beam was precisely focused at the center of the launched flyer plate.

Table S2. Summary of laser-generated flyer plate impact experiment

Thickness	Flyer Velocity (km/s)	Peak Particle Velocity u_p (km/s)	Shock Velocity u_s (km/s)	Peak Stress σ (GPa)	Density ρ (g/cm^3)
25 µm	2.5	1.7	5.2	22	3.67

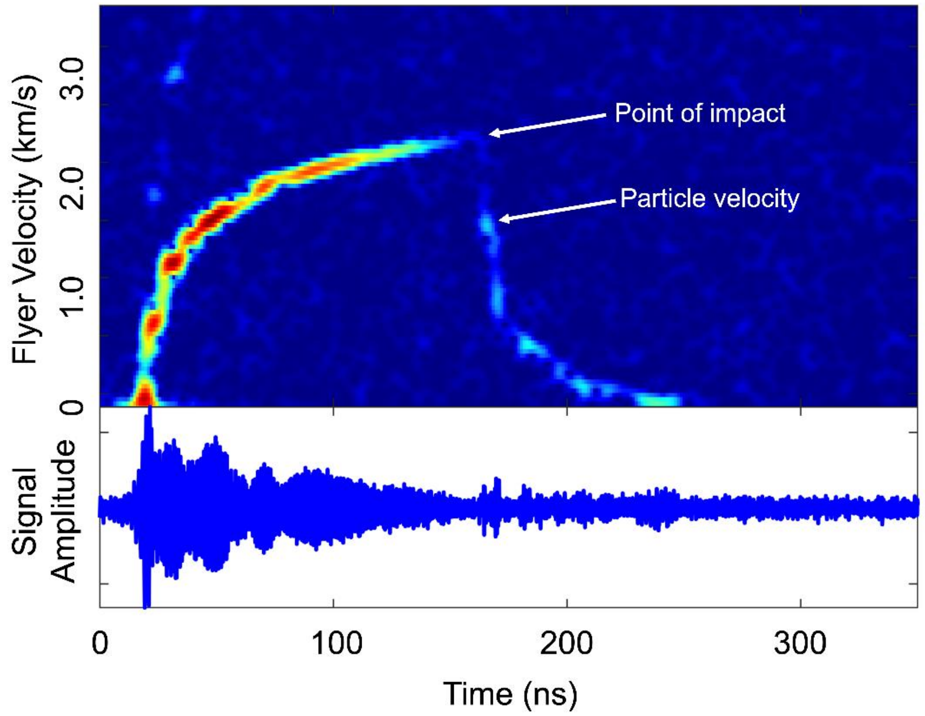

Figure S2. Representative PDV signals for flyers launched. (a) spectrogram and (b) probe signal. The point of an abrupt drop in the velocity spectrogram coincides with the flyer impact (shown via an arrow). The short duration of constant velocity (particle velocity shown via an arrow) following the flyer impact represents the period of shock generation in the sample.

The multi-scale shock technique (MSST) is an approach for simulating shock waves in compressible flow based on the Navier-Stokes equations. Unlike the direct approach, which involves simulating shock waves within large computational cells with many atoms, the MSST follows a Lagrangian point through the shock wave[3]. This allows for simulation with fewer atoms and lower computational costs. To simulate steady shock waves, the MSST uses time-evolving equations of motion for atoms and the volume of the computational cell to constrain the shock-propagation-vector stress to the Rayleigh line and energy to the Hugoniot relation. The Hugoniot relation $E - E_0 = P_0(1/\rho_0 + 1/\rho) + 0.5U^2(1 - \rho_0/\rho)^2$ is obtained by conserving mass, momentum, and energy across the shock front for a specified shock speed[4]. Here, E represents energy, P is the negative of the diagonal component of the stress tensor in the vector of the shock, U is the shock velocity, and ρ is the density. Subscripts denote the pre-shocked state, while quantities without subscripts refer to the post-shocked state. The Rayleigh line $P - P_0 = U^2\rho_0(1 - \rho_0/\rho)$ describes the thermodynamic path connecting the initial state of the system to its final (Hugoniot) state[4]. These two relations

must be satisfied for steady planar shock waves within continuum theory. Based on that, MSST method moves the system along the Rayleigh line to a shocked state.

To illustrate the effect of pressure and temperature on the crystallization process, we simulate the shocked silica glass under different pressures and temperatures. The simulations are performed following the same procedure in this study. The silica glass model consists of 8811 atoms and is obtained by the same melting-quenching procedure. As shown in Figure S3, the number of crystal atoms increases sharply within 1 ns under higher pressure and temperature (i.e., 50.5 GPa and 2730 K). However, for the low pressure and temperature (i.e., 40.2 GPa and 2550 K), the crystal atoms' number only shows a slow increase after 7 ns and the sudden increase occurs after 9 ns. Note that this pressure is close to the one obtained from the experiment [2]. The results suggest that the nucleation time is dependent on pressure and temperature. This finding helps to explain why crystallization has been observed under low pressure in experiments while it has not been observed under low pressure alone. In experiments, the time duration is much longer, which provides the glass with sufficient time for nucleation to occur despite the low mobility of atoms.

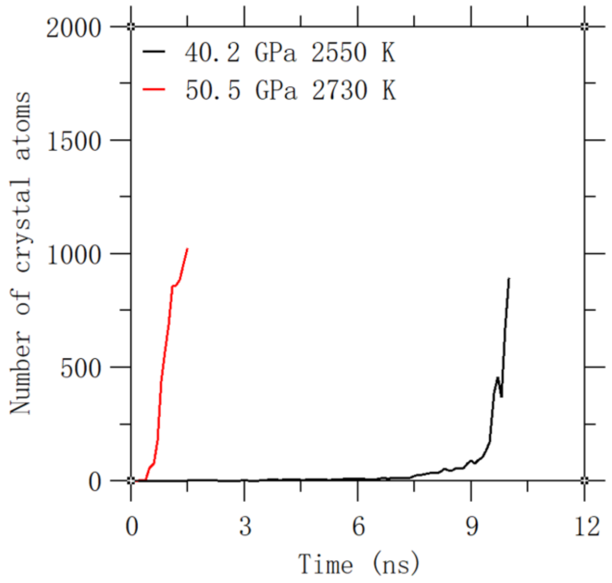

Figure S3. The evolution of the number of crystal atoms under different pressures and temperatures.

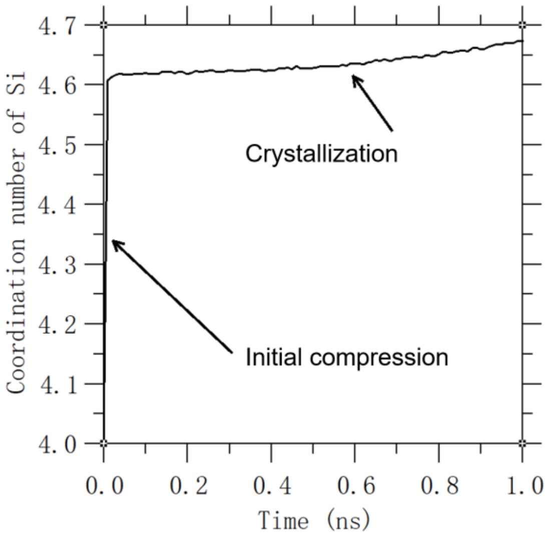

Figure S4. The global averaged coordination number of Si as the function of time. The coordination number of Si exhibits a sharp increase at the initial compression stage and a slow increase during the crystallization process.

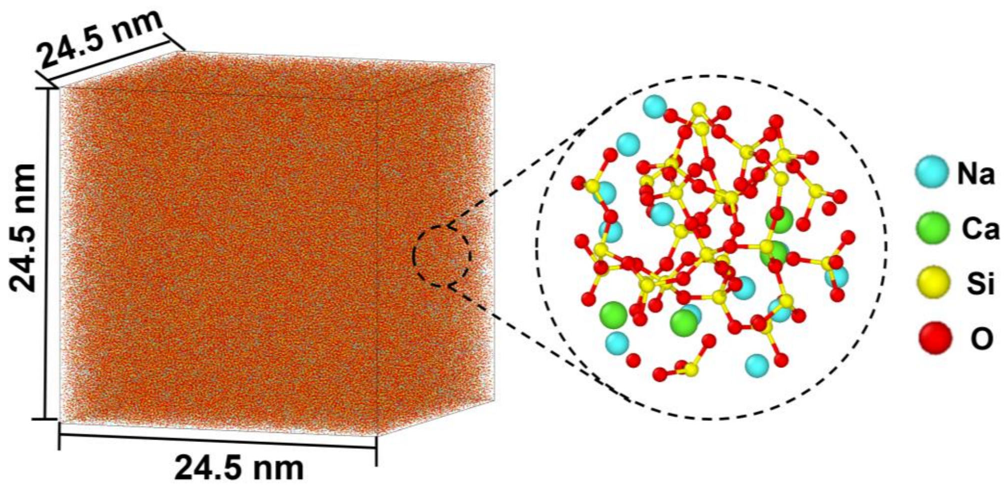

Figure S5. Schematic diagrams of the simulated large-scale atomic configuration.

Reference

(1) Sugiyama, M.; Endo, S.; Koto, K. The Crystal Structure of Stishovite under Pressure up to 6 GPa. *Mineralogical Journal* **1987**, *13* (7), 455–466. https://doi.org/10.2465/minerj.13.455.

(2) Tracy, S. J.; Turneaure, S. J.; Duffy, T. S. In Situ X-Ray Diffraction of Shock-Compressed Fused Silica. *Phys. Rev. Lett.* **2018**, *120* (13), 135702. https://doi.org/10.1103/PhysRevLett.120.135702.

(3) Reed, E. J.; Fried, L. E.; Joannopoulos, J. D. A Method for Tractable Dynamical Studies of Single and Double Shock Compression. *Phys. Rev. Lett.* **2003**, *90* (23), 235503. https://doi.org/10.1103/PhysRevLett.90.235503.

(4) Goldman, N.; Reed, E. J.; Kuo, I.-F. W.; Fried, L. E.; Mundy, C. J.; Curioni, A. Ab Initio Simulation of the Equation of State and Kinetics of Shocked Water. *J. Chem. Phys.* **2009**, *130* (12), 124517. https://doi.org/10.1063/1.3089426.
